# Supplementary material for: Graph-based pangenome analysis uncovers structural and functional impacts of allopolyploidization events
Source: Sci Adv. 2026 Oct 30;12(44):eaea5812. doi: 10.1126/sciadv.aea5812 (PMC13721995; doi:10.1126/sciadv.aea5812)
Supplement: Supplementary file 1 [file sciadv.aea5812_sm.pdf]

Science Advances
AAAS

Supplementary Materials for

Graph-based pangenome analysis uncovers structural and functional impacts of allopolyploidization events

Victor Loegler *et al.*

Corresponding author: Joseph Schacherer, jschacherer@cicbiogune.es

Sci. Adv. **12**, eaea5812 (2026)
DOI: 10.1126/sciadv.aea5812

The PDF file includes:

Figs. S1 to S9
Legends for tables S1 to S6

Other Supplementary Material for this manuscript includes the following:

Tables S1 to S6

Supplementary figures

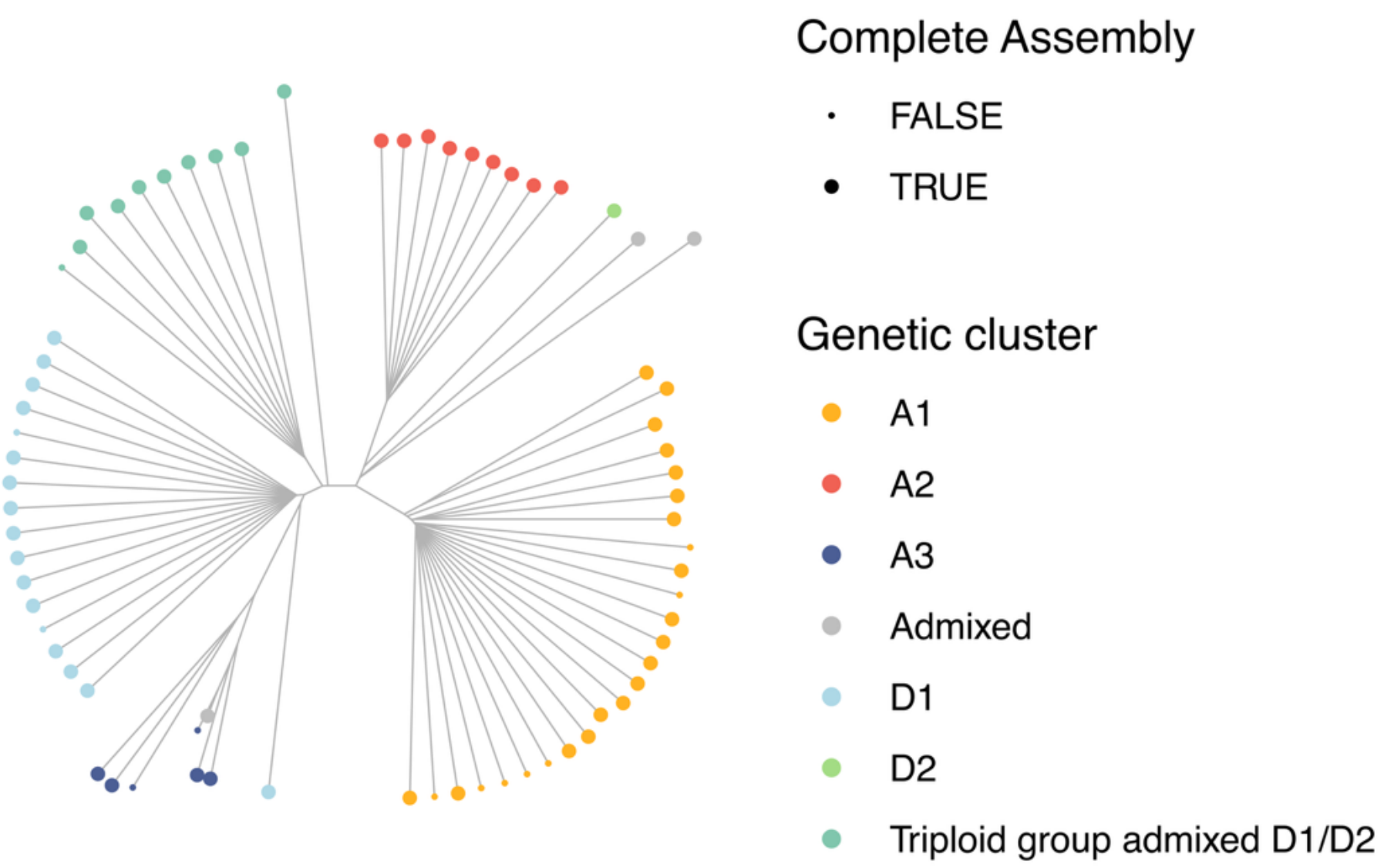

Figure S1 | Neighbor-joining tree of the 71 *B. bruxellensis* isolates built using primary subgenome reads only. Data were obtained from ref (*23*). Colors correspond to the genetic clusters define in ref (*23*). Assembly completion corresponds to a fixed threshold of 8Mb for both primary subgenome assembly and acquired subgenome assembly for allopolyploids, as defined in the method section.

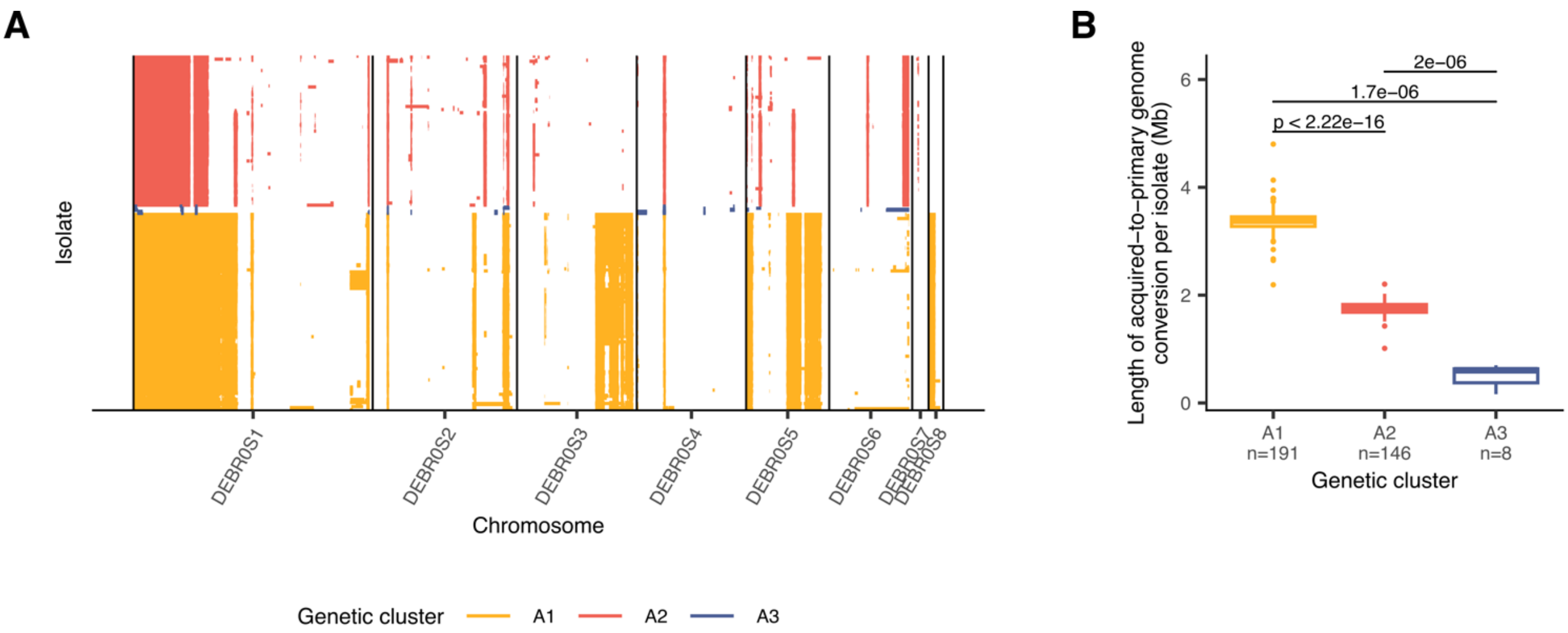

Figure S2 | Acquired-to-primary subgenome conversion events. A. Regions of acquired-to-primary subgenome conversion per isolate. The genetic cluster is color-coded. **B.** Cumulative length regions with acquired-to-primary subgenome conversion per isolate. The middle bar of the box plots corresponds to the median; the upper and lower bounds correspond to the third and first quartiles, respectively. The whiskers correspond to the upper and lower bounds 1.5 times the interquartile range (IQR). The *P* values were computed using a two-sided rank-based Wilcoxon test without further adjustment.

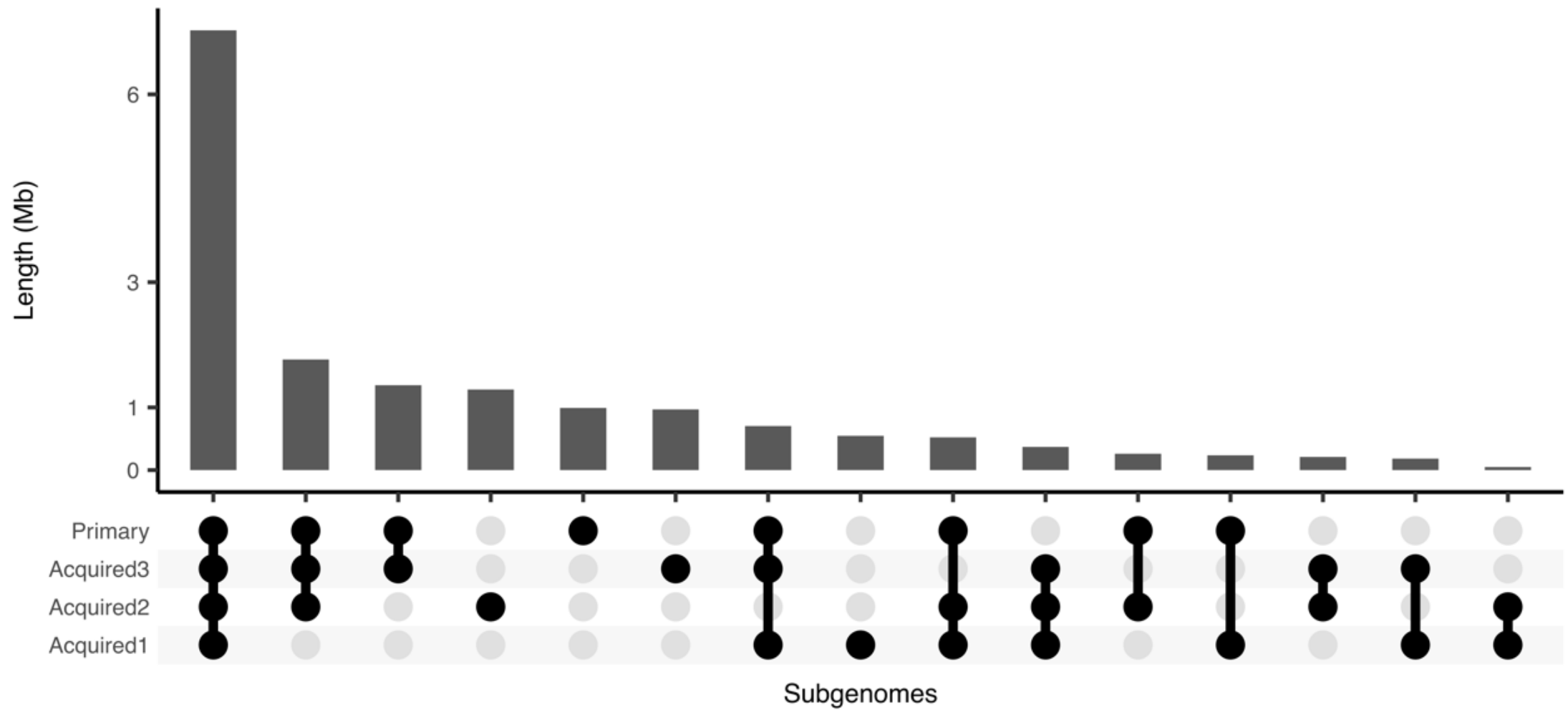

Figure S3 | Upset plot representing the cumulated length of shared segments across subgenome graphs.

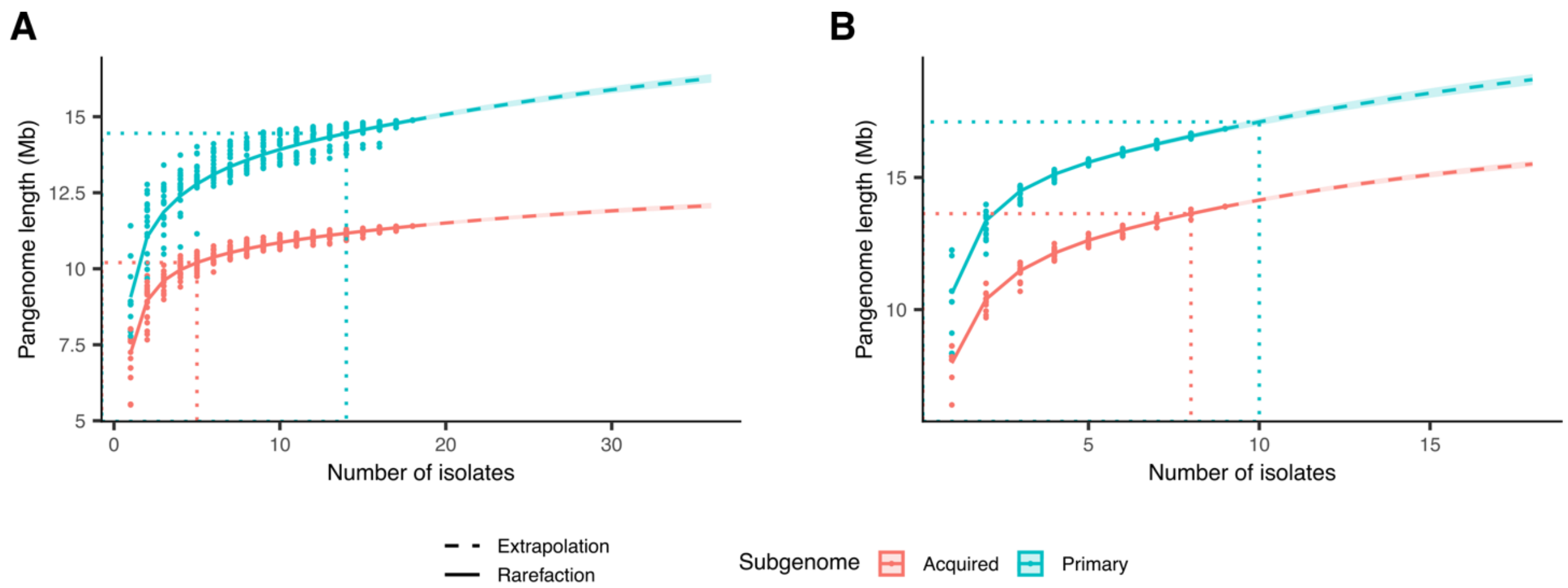

Figure S4 | Subgenome rarefaction curves. A. Rarefaction curves for primary and acquired subgenomes among A1 isolates. **B.** Rarefaction curves for primary and acquired subgenomes in A2 isolates. The points represent observed rarefaction, the curves correspond to the non-parametric rarefaction model. The dotted lines indicate the pangenome length and number of isolates required to reach 80% of the pangenome saturation.

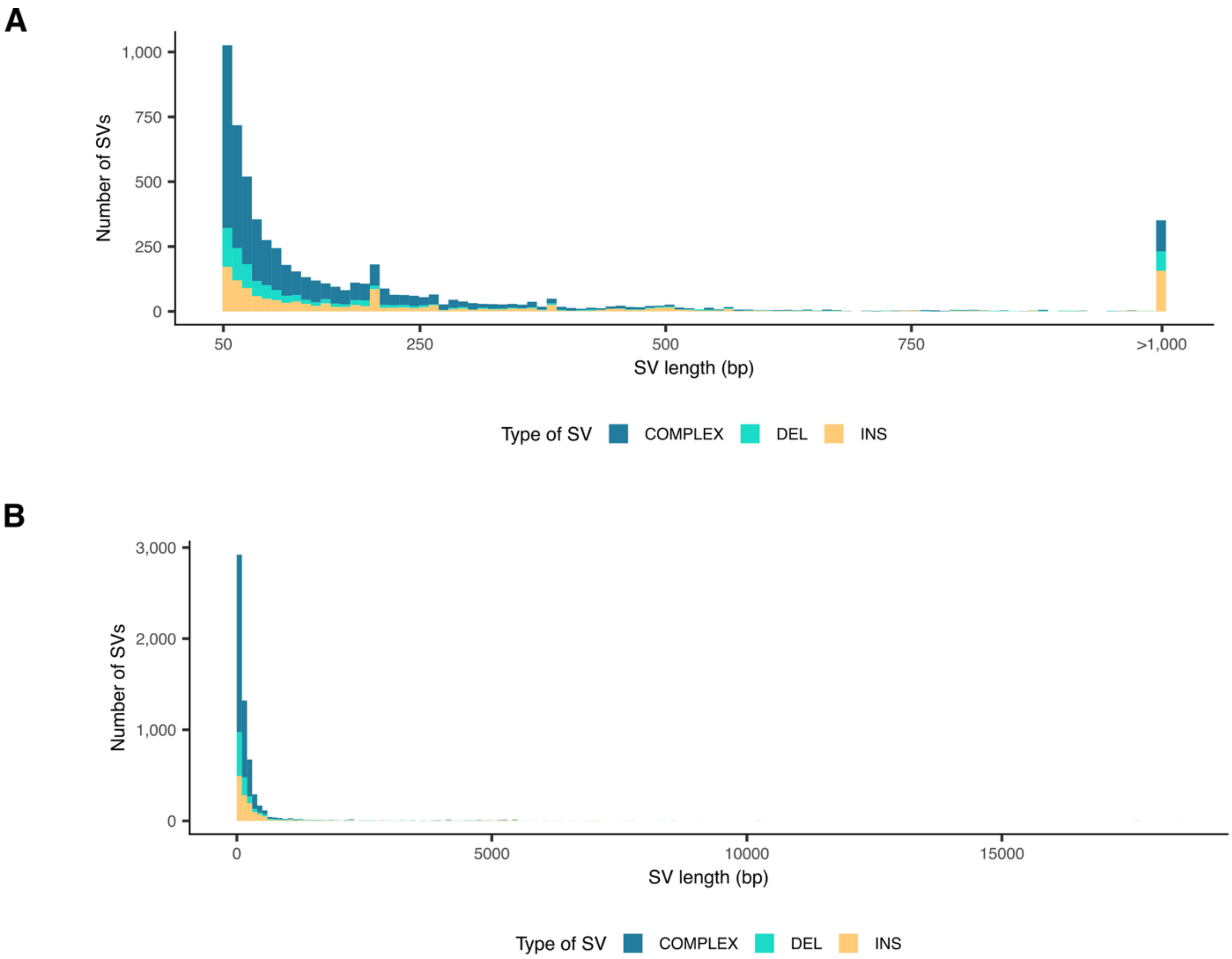

Figure S5 l SV length distribution. A. Length distribution of SVs between 50 and 1,000 bp. **B.** Global length distribution across all SVs. The type of SV is color-coded.

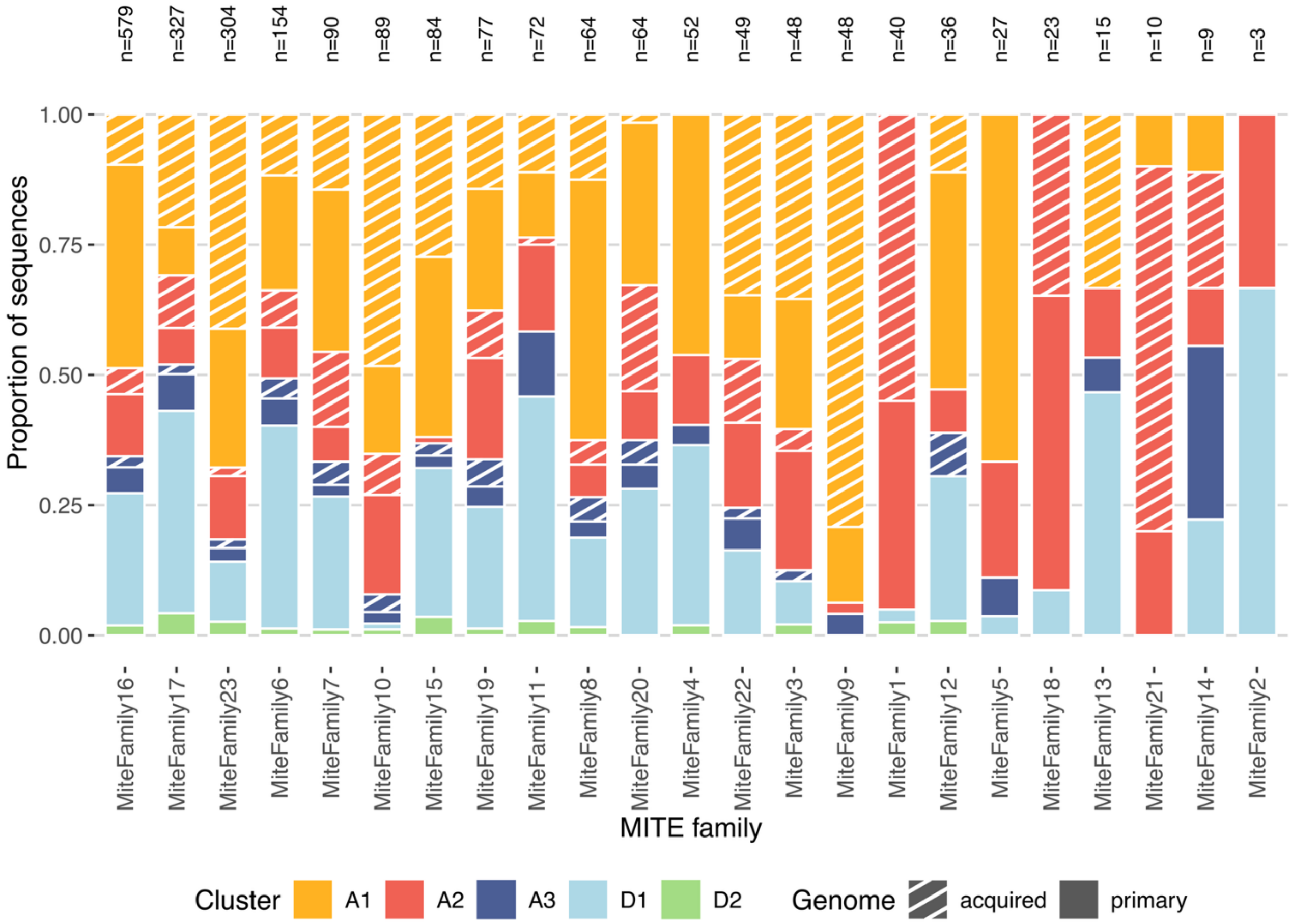

Figure S6 | Distribution of MITE families across subgenomes and genetic clusters. For each MITE family, the bar shows the proportion of sequences originating from the acquired and primary subgenomes (striped and solid patterns), further broken down by genetic cluster of origin (color-coded).

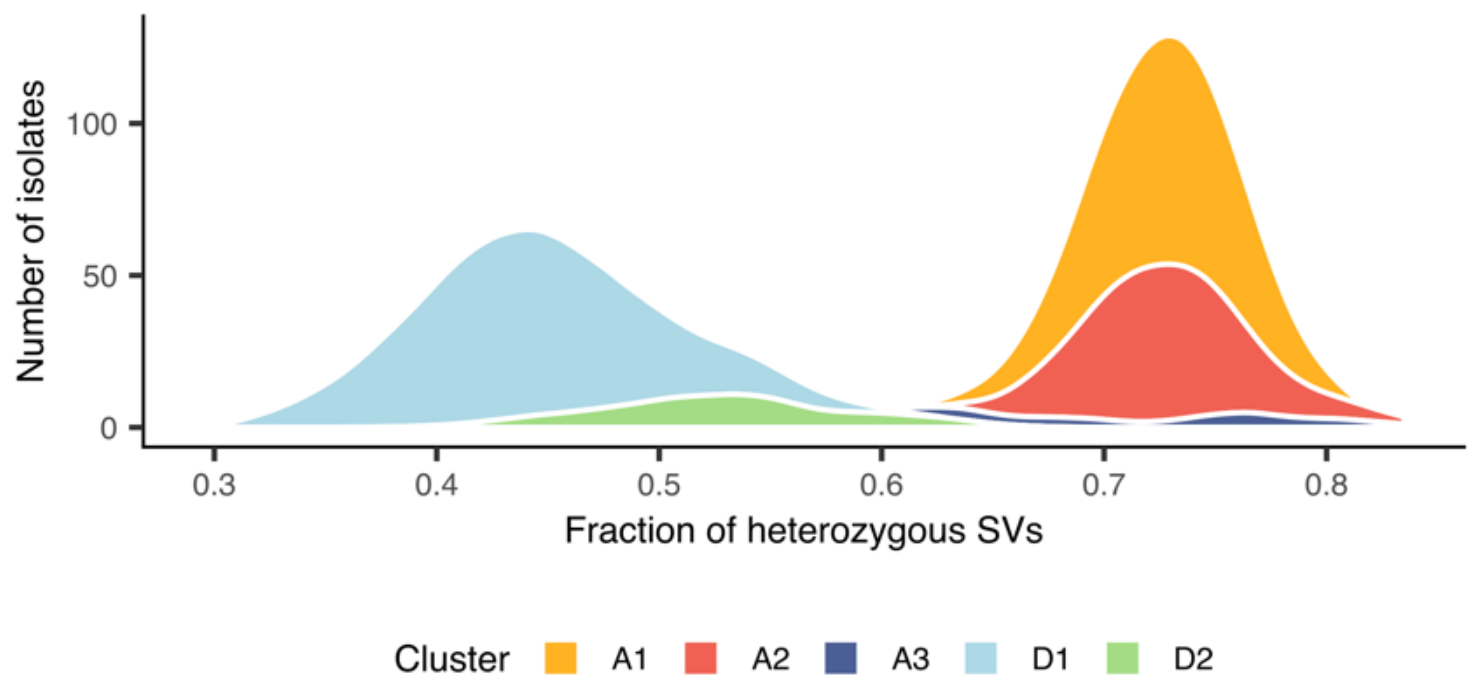

Figure S7 | Distribution of the proportion of heterozygous SVs per isolate. Genetic clusters are color-coded.

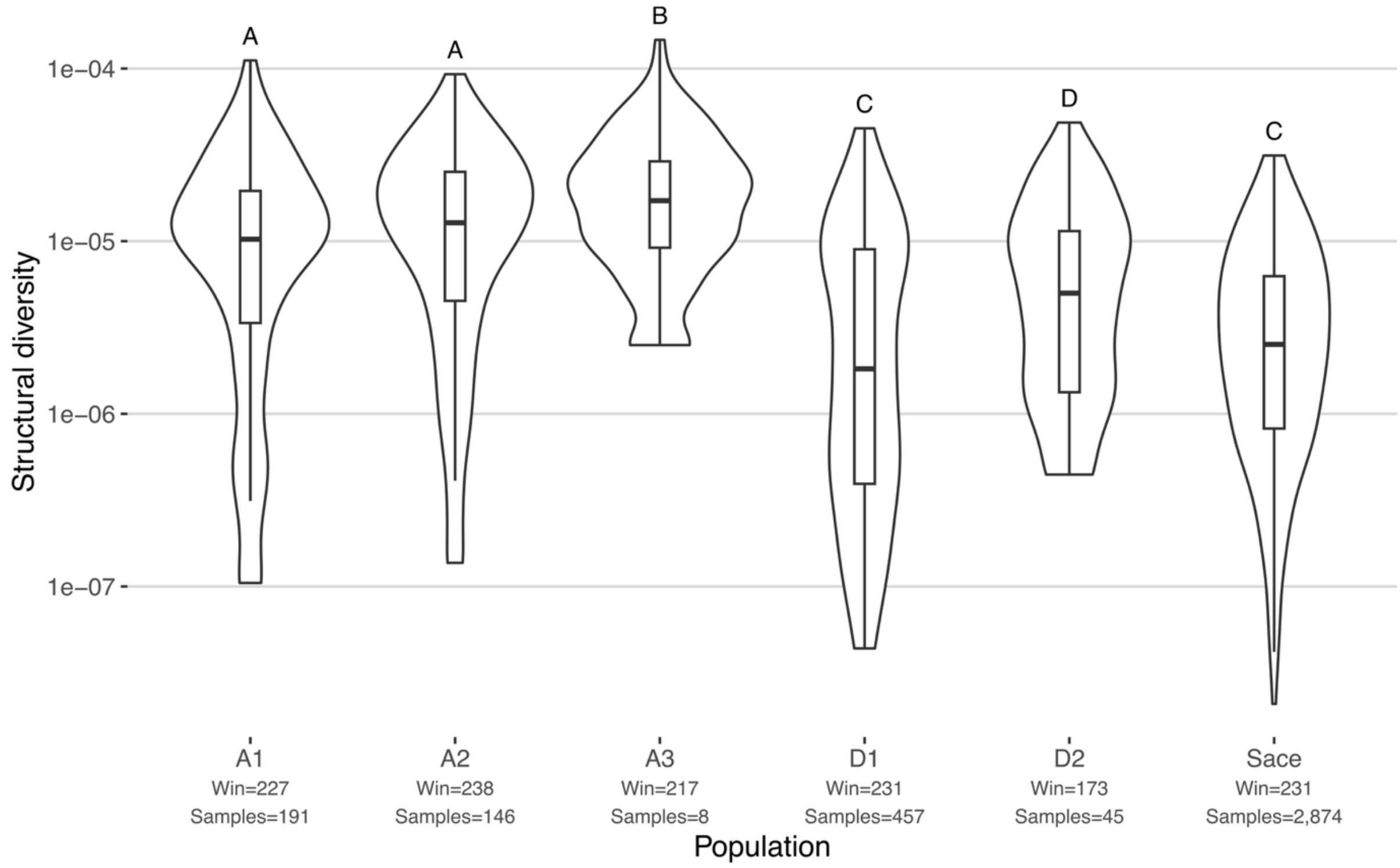

Figure S8 | Structural diversity of *S. cerevisiae* and each *B. bruxellensis* genetic cluster. Structural diversity was computed across non overlapping 50kb windows. The number of windows and samples used for each group is indicated on the x axis. The middle bar of the box plots corresponds to the median; the upper and lower bounds correspond to the third and first quartiles, respectively. The whiskers correspond to the upper and lower bounds 1.5 times the interquartile range (IQR). Letters discriminate groups between which a two-sided Mann-Whitney-Wilcoxon test with bonferroni correction is significant with P < 0.05. P-values are indicated in table S4.

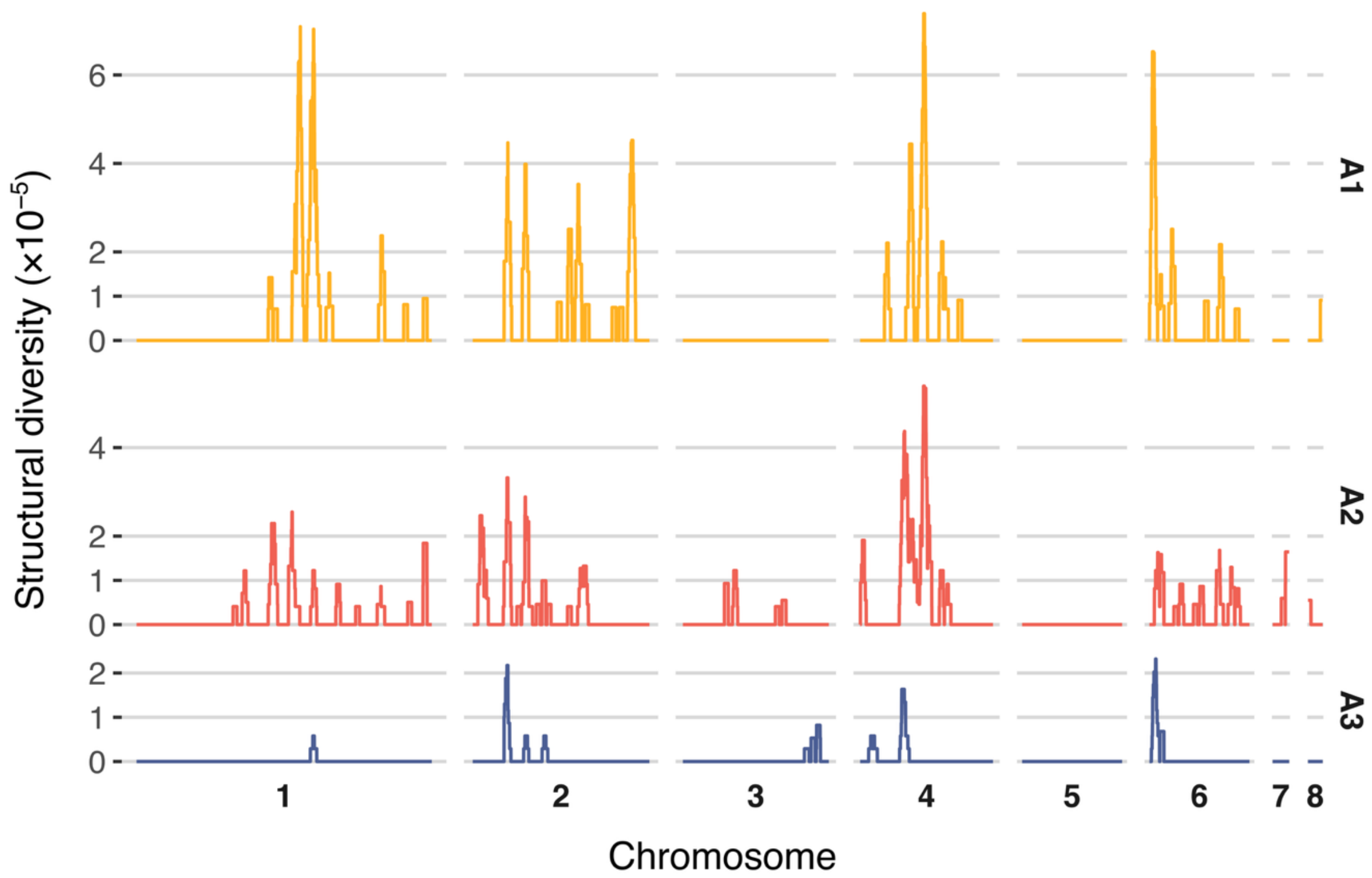

Figure S9 | Distribution of the structural diversity along the genome using hybridization-associated SVs identified in the A1, A2, and A3 genetic clusters.

Titles and captions of the supplementary tables

Table S1 I Observed and estimated length of complete and subgenome pangenomes.

Table S2 I Length of shared or private segments between subgenome-specific graphs.

Table S3 I Length of non-reference sequences per isolate and subgenome.

Table S4 I Pairwise comparisons of structural diversity between *S. cerevisiae* and each *B. bruxellensis* genetic cluster. Structural diversity was computed across 50 kb sliding windows. For each pair, the table reports the two-sided Mann-Whitney-Wilcoxon test statistic, the raw P-value, and the Bonferroni-corrected P-value. Statistical groupings shown in Fig. S8 are derived from these comparisons.

Table S5 I Hotspots of structural diversity along the Brettanomyces bruxellensis genome.

Table S6 I SV-gene pairs associations based on a linear mixed model regression.
